# Risk factors of stillbirths in four district hospitals on Pemba Island, Tanzania: a prospective cohort study

**DOI:** 10.1186/s12884-023-05613-6

**Published:** 2023-04-26

**Authors:** Tine Bruhn Skytte, Charlotte Carina Holm-Hansen, Said Mouhammed Ali, Shaali Ame, Jil Molenaar, Gorm Greisen, Anja Poulsen, Jette Led Sorensen, Stine Lund

**Affiliations:** 1 Global Health Unit, Department of Paediatrics and Adolescent Medicine, University Hospital Copenhagen, Copenhagen, Denmark; 2 Public Health Laboratory, Ivo de Carneri, Pemba, Tanzania; 3 Reproductive and Maternal Health Research Group, Public Health Department, Institute of Tropical Medicine Antwerp, Antwerp, Belgium; 4 Family Medicine and Population Health (FAMPOP), Faculty of Medical Sciences, University of Antwerp, Antwerp, Belgium; 5 Department of Neonatology, Juliane Marie Center, University Hospital Copenhagen, Copenhagen, Denmark; 6 Juliane Marie Centre for Children, Women and Reproduction, University Hospital Copenhagen, Copenhagen, Denmark; 7 Department of Clinical Medicine, Faculty of Health and Medical Sciences, University of Copenhagen, Copenhagen, Denmark

**Keywords:** Maternal health, Global health, Stillbirth, Quality of care, Intrapartum care, Low-and-middle-income country

## Abstract

**Background:**

More than 2 million third-trimester stillbirths occur yearly, most of them in low- and middle-income countries. Data on stillbirths in these countries are rarely collected systematically. This study investigated the stillbirth rate and risk factors associated with stillbirth in four district hospitals in Pemba Island, Tanzania.

**Methods:**

A prospective cohort study was completed between the 13th of September and the 29th of November 2019. All singleton births were eligible for inclusion. Events and history during pregnancy and indicators for adherence to guidelines were analysed in a logistic regression model that identified odds ratios [OR] with a 95% confidence interval [95% CI].

**Results:**

A stillbirth rate of 22 per 1000 total births in the cohort was identified; 35.5% were intrapartum stillbirths (total number of stillbirths in the cohort, n = 31). Risk factors for stillbirth were breech or cephalic malpresentation (OR 17.67, CI 7.5-41.64), decreased or no foetal movements (OR 2.6, CI 1.13–5.98), caesarean section [CS] (OR 5.19, CI 2.32–11.62), previous CS (OR 2.63, CI 1.05–6.59), preeclampsia (OR 21.54, CI 5.28–87.8), premature rupture of membranes or rupture of membranes 18 h before birth (OR 2.5, CI 1.06–5.94) and meconium stained amniotic fluid (OR 12.03, CI 5.23–27.67). Blood pressure was not routinely measured, and 25% of women with stillbirths with no registered foetal heart rate [FHR] at admission underwent CS.

**Conclusions:**

The stillbirth rate in this cohort was 22 per 1000 total births and did not fulfil the *Every Newborn Action Plan’s* goal of 12 stillbirths per 1000 total births in 2030. Awareness of risk factors associated with stillbirth, preventive interventions and improved adherence to clinical guidelines during labour, and hence improved quality of care, are needed to decrease the stillbirth rate in resource-limited settings.

**Supplementary Information:**

The online version contains supplementary material available at 10.1186/s12884-023-05613-6.

## Background

Stillbirths are a global challenge, with approximately 2 million third-trimester stillbirths occurring every year [1]. It has been estimated that 42.3% of intrapartum stillbirths can be prevented with quality care at birth [1]. The vast majority of all stillbirths occur in low- and middle-income countries, and two-thirds occur in sub-Saharan Africa and South Asia alone [1, 2].

The Millennium Development Goal 4 aimed to reduce the under-5-year and infant mortality. The under-5-year mortality rate has declined, but the neonatal and stillbirth rates are yet to follow this reduction in mortality. In the post-2015 era, the Sustainable Development Goals do not have specific targets for stillbirths [2]. The Every Newborn Action Plan targets a stillbirth rate lower than 12 per 1000 total births in every country by 2030, requiring a 4.2% reduction in the global stillbirth rate per year [2]. Since 2005, national-level estimates on causes of neonatal death have been available, but with no similar information on stillbirths. There is thus a lack of data on the underlying causes of stillbirths [1–3].

Stillbirth rates are generally a good indicator of the quality of care before and during childbirth. The period from the onset of labour until birth is the most high-risk period for the mother and child, where 45% of all stillbirths occur [4]. Sound quality of care during pregnancy and childbirth is a crucial factor in lowering the stillbirth rate in countries where the burden remains high. Interventions focusing on family planning, antenatal care and skilled birth attendants assisting during childbirth have been proven to lower the stillbirth rate [3].

Previous studies and systematic reviews identified risk factors associated with stillbirth in low- or middle-income settings, including, but not limited to, maternal age, parity, gestational diabetes, maternal hypertensive disorders, antenatal care visits, education level and birth presentation [5–8]. Data from high-income countries suggest that the stillbirth rate can be lowered with adequate care, necessary equipment and interventions during childbirth [9–11].

Tanzania is among the top ten countries with the highest numbers of stillbirths [5]. In 2019, the stillbirth rate for Tanzania was 18.8 per 1000 total births [12, 13]. The Zanzibar archipelago previously did not collect data on stillbirths, yet since 2019 stillbirths have started to be routinely recorded and reported [12]. Few studies have investigated the stillbirth rate, risk factors and underlying causes in the Zanzibar archipelago [6, 14]. Most studies on stillbirths and risk factors in low-income settings are carried out in tertiary, university or referral hospitals and therefore do not represent the large number of births in district hospitals in more rural settings. Furthermore, many studies are retrospective and based on data retrieved from case files, representing challenges with recall bias and selective data output respectively [15, 16].

We present a prospective cohort study on four district hospitals in a resource-limited setting, Pemba Island, Zanzibar. To our knowledge, this is the first prospective cohort study on stillbirths at four secondary-level hospitals on this scale globally.

This study aims to determine stillbirth incidence and identify the risk factors for stillbirth and quality of care during childbirth in this setting.

## Methods

### Study design

We used a prospective cohort study design nested within the baseline of a pre-post intervention study, the Newborn Emergency Outcome-Study, NEO-study (clinicaltrial.gov NCT040937778) to reduce neonatal mortality. Data was collected over eleven weeks, from the 13th of September until the 29th of November 2019.

### Setting

Pemba is an island in the Zanzibar archipelago. There are four district hospitals on the island, and they assist a total of approximately 11,000 births each year [12]. Pemba has approximately 450,000 inhabitants [17] and is a predominantly rural setting [6, 17]. Low rates of epidemic diseases are reported in Pemba. Malaria is almost eradicated, with only 12 cases in pregnant women in 2019 and zero malaria-related fatalities reported in the same year [12]. HIV is tested among 98.9% of women attending antenatal care [ANC] visits, and a positivity rate of 0.04% was reported in 2019. The overall syphilis testing rate is 21% on Pemba, and the positivity rate was 0.22% in 2019 [12]. Hospitals are anonymised in this study to protect and respect the healthcare workers’ confidentiality. See Appendix 1 for a detailed view of the setting.

In 2019 two hospitals were labelled as district hospitals and one as a regional hospital, while Micheweni Hospital was in the process of being accepted as a district hospital. By the time the study was conducted, CS was possible, and a medical doctor was on call at night at Micheweni Hospital. We, therefore, included Micheweni hospital as a district hospital since it was capable of maternal, childbirth and newborn care at the same level as the other three hospitals. Mkoani hospital (Abdulla Mzee Hospital) was run in collaboration between ChinaAid and the Zanzibar Government. Chinese doctors routinely did rotations in the hospital for six months at a time. The hospital was labelled a Regional Hospital in the Zanzibar Health bulletin 2019 but operated similarly to the other hospitals on Pemba. The overall facility rate for births is increasing in Pemba, with 67.6% of all births occurring in health facilities in 2019 [12]. All hospitals have access to instrumental assisted delivery such as forceps, vacuum extraction and caesarean section. Human resources are scarce; only a few medical doctors or clinical officers are available at each hospital to cover all medical specialities. Clinical officers operate as doctors and have completed three years of medical education where medical doctors have five years of medical education and one year of internship [18, 19]. Generally, midwives handled the maternal and childbirth wards. There were a prenatal, childbirth and postnatal wards in all hospitals. Typically 3–4 midwives were at work during the day and took care of all three wards. At night, usually only 1–2 midwives took care of the wards. Due to the shortage of staff, midwives often faced multiple childbirths to handle at one time. At night medical doctors were on call from home. Emergency ambulances are available but are rarely used. The only tertiary hospital in the Zanzibar Archipelago was located at Unguja (the main island) and served as a referral hospital. In reality, referrals were scarcely done since the ferry took 6–8 h to Unguja, and travel by aeroplane was financially unavailable for most patients. Few referrals between the hospitals on Pemba were made; e.g. when a doctor at another hospital had more experience in a specific procedure, sometimes a patient was moved, yet this was a rare occasion. Information on midwives’ workload and organisation of the maternity ward is based on the authors’ observations prior and during the study period. Data on referrals from smaller facilities and records of where the woman was admitted from was rarely available, thus not collected in this cohort.

### Eligible criteria

All women giving birth at one of Pemba’s four district hospitals were eligible for participation in the NEO-study.

All singleton stillbirths with gestation age [GA] > 28 weeks or birth weight [BW] > 1000 g were eligible for inclusion. Stillbirths were defined as babies not showing any signs of life after birth [20]. The World Health Organization [WHO] classification system was used to discriminate between miscarriages or abortions and stillbirths (GA > 28 weeks or BW > 1000 g) in line with Lawn et al. [2]. Foetal heart rate at admission was used to discriminate between antepartum and intrapartum stillbirths.

**Fig. 1:**
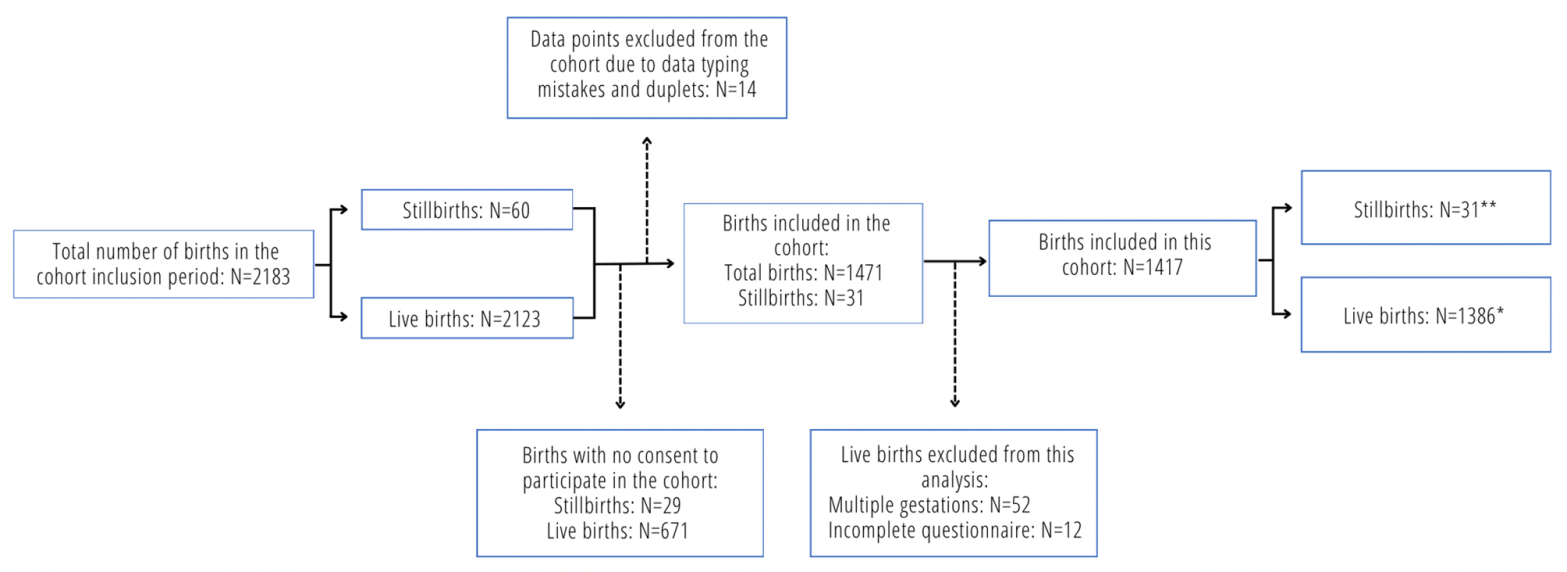
Inclusion of births in the cohort. The stillbirth rate was 21 stillbirths per 1000 total births. *Live births: All singleton live births, **Stillbirths: All singleton births not showing any signs of life after birth and birth weight > 1000 g. or GA > 28 weeks

In the reference group, all singleton live births were eligible for inclusion in the cohort. See Fig. 1.

Exclusion criteria was absence of consent from the woman giving birth prior to the birth.

Births from multiple gestations were excluded from this study because data was often too ambiguous, e.g. only data from one twin was available. Additionally, this study had no multi-gestational births in the stillbirth group. Data from the cohort, including multi-gestational births, will be reported in a separate paper.

### Patient and public involvement statement

This study did not involve patients or the public in the design, conduct, reporting or dissemination of the study findings.

### Informed consent, data collection and management

18 research assistants with a medical background (midwives, nurses or clinical officers) were trained in data collection skills and the use of REDCap on data collection tablets. Research assistants were present in the maternity and delivery ward 24 h a day. They approached all women as soon as possible after admission and no later than the expulsion phase of the second stage of labour. Informed consent to the NEO-study, including a motion-triggered video recording of the newborn and post-natal questionnaire, was given in a written form and could be retrieved at any time. If the woman was too far in labour or in too much pain, it was not attempted to retrieve consent. After delivery, consent could be obtained for participating only in the questionnaire part of the study. All health workers in the maternity units of the four hospitals consented to participate in the study.

The mothers were approached after childbirth to confirm consent and fill out a postnatal questionnaire about sociodemographic characteristics, obstetric history, and obstetric risk factors before and during childbirth. The healthcare worker in charge of the specific birth was approached after the birth and filled out a questionnaire about the birth, obstetric risk factors and birth outcome. Medical records could be used if a health worker was in doubt about the answer to a question, e.g. regarding previous medical and obstetric background. The medical records were not routinely used in the data collection though, since not all women giving birth had an updated record or information was unreadable. All childbirths were registered in a register book with an overview of each woman and child, including information on the mother’s age, birthweight, outcome (e.g. live birth or stillbirth), parity, delivery method, and a possibility for an extra remark on the childbirth. The health worker could also use this register to help answer to gather informaton. All questionnaires were filled out with the guidance of a research assistant. Research assistants also collected pictures of each woman’s partograph. Data were collected and managed using REDCap (v5.12.1) electronic data capture tools hosted and stored securely at the Capital Region of Denmark (RegionH). Lenovo 7 tablets were used for data collection. Data were exported to SPSS version 28.0.0.0 and analysed. Hospital registers were used for crosschecking in cases where reported BW values were considered unlikely. Video data from the NEO-study is currently in review for publishing, and one article is already published [21].

### Statistical analysis

Sample size calculation was not performed for this study since it was a secondary outcome in the Newborn-Emergency-Outcome-study. Thus, the study was based on recruitment duration rather than the sample size. The stillbirth rate was based on the stillbirth incidence in the cohort. It was calculated with the number of stillbirths in the cohort divided with the number of total births in the cohort. Since not all births were included in the cohort, we calculated the stillbirth rate for all births in the study time and for the births included in the cohort.

Descriptive analysis showed frequencies and distribution of the variables. Means of continuous variables were compared using independent samples t-test. Fischer’s exact test or Pearson Chi-Square test was performed for categorical variables to test for significant differences in categorical variables between stillbirths and live births. A binomial logistic regression model generated odds ratios [OR] for the variables analysed. Since the outcome analysed is below 10% of the reference group, the OR provides a reasonable approximation of the relative risk [22]. P-values < 0.05 were considered statistically significant.

### Ethical approval and consent to participate

Ethical approval was obtained from the Zanzibar Health Research institute ethical committee, Ministry of Health Zanzibar (NO.ZAHREC/02/August/2019/30). All methods were carried out in accordance with relevant guidelines and regulations (Declaration of Helsinki).

## Results

During the study period, 2183 births were eligible for participation, of which 60 were stillbirths (Fig. 1). Of the eligible participants, 1452 women were accepted for enrolment. Seventy-eight were excluded due to multi-gestation pregnancy, incomplete questionnaires, typing mistakes or duplicate records. Of the remaining included 1417 births, 1386 were live births, and 31 were stillbirths. The stillbirth rate was 22 stillbirths per 1000 total births in the cohort (Fig. 1).

Overall, there was no significant sociodemographic difference between women with stillbirths and live births, see Table 1. Most women were married, approximately one-third had no formal or primary education, and the dominant religion was Islam. Partographs were collected but proved not to be used routinely in this setting. Partographs were available for only 39% (551/1417) of the included births; they typically contained little information, had poor photo quality, and further analysis was not attempted.

Data showed that women were significantly more likely to give birth to a stillborn baby at Hospital 1 (OR 3.11, 95% CI 1.17–8.28) than those giving birth at Hospital 4, see Table 1.

**Table 1:** Sociodemographic characteristics of the women giving birth

	Live births, n = 1386	Stillbirths, n = 31	OR (95% CI)	P-value
**Age in years**				0.623^a^
Mean (Std. deviation)	26.47 (± 6.35)	25.90 (± 5.75)	0.99 (0.93–1.05)	
Median	25	26		
Missing	8	0		
**Living district**				0.081*
Chake Chake	449 (32.8%)	7 (23.3%)	Ref	
Micheweni	250 (18.3%)	10 (33.3%)	2.56 (0.97–6.82)	
Wete	354 (25.9%)	4 (13.3%)	0.73 (0.21–2.5)	
Mkoani	315 (23%)	9 (30%)	1.83 (0.68–4.97)	
Missing	18	1		
**Hospital**				0.037*
Hospital 1	521 (37.7%)	7 (22.6%)	**3.11 (1.17–8.28)**	
Hospital 2	321 (23.2%)	4 (12.9%)	0.93(0.27–3.19)	
Hospital 3	301 (21.8%)	10 (32.3%)	2.47 (0.93–6.56)	
Hospital 4	239 (17.3%)	10 (32.3%)	Ref	
Missing	4	0		
**Education**				0.350**
No formal education or primary	439 (32%)	12 (40%)	1.42 (0.68–2.97)	
Secondary, college and above	935 (68%)	18 (60%)	Ref	
Missing	12	1		
**Marital status**				0.393*
Married	1356 (98.4%)	29 (96.7%)	Ref	
Unmarried	22 (1.6%)	1 (3.3%)	2.13 (0.28–16.31)	
Missing	8	1		

*Note. OR* Odds ratio, *CI* Confidence interval

*Fischer’s exact test, **Pearson Chi-Square test, ^a^Independent samples t-test. Significant results are in bold

### Antepartum history and events

Women presenting with a history of previous CS proved to be more at risk of stillbirth (OR 2.63, 95% CI 1.05–6.59), Table 2. Almost all women attended ANC visits at least once. The number of ANC visits did not differ significantly between women giving birth to stillbirths and live births. Women with preeclampsia had a significantly higher risk of stillbirth (OR 21.54, 95% CI 5.28–87.8).

**Table 2:** Antepartum history of the women giving birth divided by stillbirths and live births

	Live births, n = 1386	Stillbirths, n = 31	OR (95% CI)	P-value
Pregnancies*				0.275***
Primiparous (1)	376 (27.2%)	12 (38.7%)	2.03 (0.85–4.85)	
Multiparous (2–4)	571(41.3%)	9 (29%)	Ref	
Grandparous (> 5)	435(31.5%)	10 (32.3%)	1.46(0.59–3.62)	
Missing	4	0		
History of miscarriages and/or abortion*				0.592***
Yes	231 (16.9%)	6 (20.7%)	1.28 (0.52–3.18)	
No	1135 (83.1%)	23 (79.3%)	Ref	
Missing	20	2		
Previous CS*				0.032***
Yes	123 (9%)	6 (20%)	**2.63 (1.05–6.59)**	
No	1242 (91%)	23 (76.7%)	Ref	
Missing	22	1		
ANC visits attended*				0.103**
Yes	1368 (99.7%)	29 (96.7%)	Ref	
No	4 (0.3%)	1 (3.3%)	**11.79 (1.28–108.8)**	
Missing	14	1	0	
ANC visits*				0.710***
1–3 visits	705 (51.8%)	14 (48.3%)	0.87 (0.42–1.82)	
> 4 visits	657 (48.2%)	15 (51.7%)	Ref	
Missing	24	2		
Preeclampsia*				< 0.001**
Yes	7 (0.5%)	3 (10%)	**21.54 (5.28–87.8)**	
No	1357 (99.5%)	27 (90%)	Ref	
Missing	22	1	0	

*Note. ANC* Antenatal care, *CS* caesarean section, *OR* Odds ratio, *CI* confidence interval

*Answered by mothers, **Fischer’s exact test, ***Pearson Chi-Square test. Significant results are in bold

### Intrapartum events

If the women felt decreased or no foetal movements, the risk of stillbirth was significantly higher (OR 2.6, 95% CI 1.13–5.98). Among stillbirths, 11 (35.5%) versus 1041 (76.3%) of live births had FHR registered at admission. Thus at least 11 stillbirths were considered intrapartum, Table 3. Women with foetuses in different presentation than cephalic had a significantly higher risk of stillbirth, Table 4. Women with stillbirths were more likely to have preterm premature rupture of membranes (PPROM) or rupture of membranes more than 18 h before birth (Premature rupture of membranes, PROM) compared to women with live births (OR 2.5, 95% CI 1.06–5.94), Table 4. Stillbirths were more likely to be born in meconium stained amniotic fluid (OR 12.03, 95% CI 5.23–27.67), Table 4. Women with a stillbirth has significantly higher odds of delivering by CS (OR 5.19, 95% CI 2.32–11.62), Table 4. Five out of nine of women with stillbirth and delivery with CS had no FHR at admission (55%), Table 3. Only one (0.1%) of the women with live births and none with a stillbirth had assisted childbirth with vacuum extraction or forceps, see Table 4.

**Table 3:** Foetal heart rate monitoring and adherence to guidelines

	Live births, n = 1386	Stillbirths, n = 31	OR (95% CI)	P-value
**FHR at admission registered**				<0.001***
*“Was there foetal heart rate on admission?”*				
Yes	1041 (76.3%)	11 (35.5%)	Ref	
No / Marked as unknown	324 (23.7%)	20 (64.5%)	**5.84 (2.77–12.32)**	
Missing	21	0		
**FHR value at admission**				<0.001**
Slow < 120 bpm	8 (0.6%)	2 (6.5%)	**31.5 (5.76-172.17)**	
Normal 120–160 bpm	1008 (72.7%)	8 (25.8%)	Ref	
Fast > 160 bpm	8 (0.6%)	0 (0%)	0	
No value measured	362 (26.1%)	21 (67.7%)	**7.31 (3.21–16.65)**	
Missing	0	0		
**Last measured FHR value**				<0.001**
Slow < 120 bpm	48 (3.5%)	2 (6.5%)	5.01 (0.98–25.47)	
Normal 120–160 bpm	721 (52%)	6 (19.4%)	Ref	
Fast > 160 bpm	14 (1%)	0 (0%)	0	
No value measured	603 (43.5%)	23 (74.2%)	**4.58 (1.85–11.33)**	
Missing	0	0		
**FHR value measured at any time during admission** ^a^				< 0.001**
No foetal heart rate value measured at any time	275 (19.8%)	19 (61.3%)	**6.4 (3.07–13.34)**	
Foetal heart rate value measured during admission	1111 (80.2%)	12 (38.7%)	Ref	
**Delivery mode with no foetal heart rate at admission** ^b,c^				0.006**
CS with no foetal heart rate at admission	24 (7.5%)	5 (25%)	**4.13 (1.38–12.32)**	
SVD with no FHR at admission	297 (92.5%)	15 (75%)	Ref	
Missing	3	0		

*Note. FHR* Foetal heart rate, *CS* Caesarean section, *OR* Odds ratio, *CI* Confidence interval

*Fischer’s exact test, **Pearson Chi-Square test, ^a^Computed from the variables “FHR value at admission” and “Last measured FHR value”. If no values were collected in either of the two, there was no foetal heart rate value measured at any time. ^b^Vacuum extraction or forceps was not performed in women presenting with no FHR at admission. ^c^Crosstab made with layering. “FHR at admission”-variable used for layering and “Delivery mode” used in the crosstab. Significant results are in bold

**Table 4:** Intrapartum events of women giving birth divided by stillbirths and live births

	Live births, n = 1382	Stillbirths, n = 31	OR (95% CI)	P-value
Decreased or no foetal movements*				0.020***
Yes	182 (13.4%)	8 (18.6%)	**2.6 (1.13–5.98)**	
No	1181 (86.6%)	20 (71.4%)	Ref	
Missing	23	3		
**Presentation**				<0.001***
Cephalic	1335 (97.5%)	20 (69%)	Ref	
Breech or cephalic malpresentation	34(2.5%)	9 (31.%)	**17.67 (7.5-41.64)**	
Missing	17	2		
**Oxytocin given**				0.877***
Yes	968 (70.3%)	20 (69%)	Ref	
No	409(29.7%)	9 (31%)	1.07 (0.48–2.36)	
Missing	9	2		
**Oxytocin given as labour augmentation**				0.669***
Yes	70 (5.1%)	2 (6.5%)	0.77 (0.18–3.3)	
No	1316 (94.9%)	29 (93.5%)	Ref	
Missing	0	0		
**Rupture of membranes before week 37 or 18 hours before delivery**				0.031***
Yes	149 (10.8%)	7 (23.3%)	**2.5 (1.06–5.94)**	
No	1226 (89.2%)	23 (76.7%)	Ref	
Missing	12	0		
“Did your water broke before term?* (PPROM)				0.006***
Yes	267 (19.6%)1098	12 (40%)	**2.74 (1.31–5.76)**	
No	(80.4%)	18 (60%)	Ref	
Missing	21	1		
Water broke 18 hours before delivery*				0.040***
Yes	124 (9%)	6 (20%)	**2.52 (1.01–6.28)**	
No	1249 (91%)	24 (80%)	Ref	
Missing	13	1		
**Fever before or during labour**				0.163**
Yes	7 (0.5%)	1 (3.2%)	6.53 (0.78–54.73)	
No	1371 (99.5%)	30 (96.8%)	Ref	
Missing	8	0		
High fever during labour*				0.212**
Yes	10(0.7%)1364	1 (3.3%)	0.21 (0.026–1.72)	
No	(99.3%)	29 (96.7%)	Ref	
Missing	12	1		
**Amniotic fluid was purulent or foul-smelling**				0.083***
Yes	109 (7.9%)	5 (16.7%)	2.32 (0.87–6.18)	
No	1264 (92.1%)	25 (83.3%)	Ref	
Missing	13	1		
**Antepartum hemorrhage (vaginal bleeding before delivery)**				0.052***
Yes	129 (9.4%)	6 (20%)	2.41 (0.97–5.99)	
No	1242 (90.6%)	24 (80%)	Ref	
Missing	15	1		
**Amniotic fluid**				<0.001***
Clear	1319 (96.6%)	21 (67.7%)	Ref	
Meconium	47 (3.4%)	9 (29.0%)	**12.03 (5.23–27.67)**	
Missing	20	1		
**Delivery mode**				<0.001***
Spontaneous vaginal delivery	1272 (92.3%)	21 (70%)	Ref	
Vacuum extraction/forceps	1 (0.1%)	0 (0%)	0	
Caesarean section	105 (7.6%)	9 (30%)	**5.19 (2.32–11.62)**	
Missing	8	1		

Note. FHR Foetal heart rate, PPROM Preterm prelabour rupture of membranes, OR Odds ratio, CI Confidence interval

*Answered by mothers, **Fischer’s exact test, *** Pearson Chi-Square test. Significant results are in bold

### Adherence to guidelines

Partographs were apparently neither used routinely nor efficiently. Nineteen of the mothers who gave birth to a stillborn (61.3%) had no FHR value recorded at any time. This was also the case for 275 (19.8%) of live births, Table 3. Variances in FHR or no recording of FHR during admission were found to be associated with stillbirth. It was more likely not to have any measurement of FHR during admission, if the outcome was stillbirth (OR 6.4, 95% CI 3.07–13.34). Five of 20 women giving birth to stillbirths with no (or marked as unknown) registered FHR at admission were delivered by CS, see Table 3. Blood pressure was not measured routinely: 19 (61.3%) of women giving birth to a stillbirth and 885 (64.4%) of women giving birth to a live birth had no record of blood pressure measurement, Table 5.

**Table 5:** Blood pressure measurement and adherence to guidelines

	Live births, n = 1386	Stillbirths, n = 31	OR (95% CI)	P-value
**Blood pressure of mother measured**				0.990**
Yes	489 (35.6%)	11 (35.5)	Ref	
No / marked as unknown	885 (64.4%)	20 (64.5%)	1.01 (0.48–2.11)	
Missing	12	0		

*Note. FHR* foetal heart rate, *CS* caesarean section, *OR* Odds ratio, *CI* confidence interval

*Fischer’s exact test, **Pearson Chi-Square test. Significant results are in bold

## Discussion

This prospective cohort study found a stillbirth rate of 22 per 1000 total births in the cohort and a stillbirth rate of 27.5 per total births for all births in the study period in a low-resource setting on Pemba in Zanzibar, Tanzania. In 11 cases, foetal heart beating was heard on admission. In further 19 cases, FHR was not recorded at any time, and some of these fetuses may also have been alive at admission. We identified previous CS, preeclampsia, hospital location, decreased or no foetal movements, slow FHR or no recorded FHR at any time, breech and cephalic malpresentation, premature rupture of membranes (PPROM), rupture of membranes 18 h before birth (PROM) and meconium-stained amniotic fluid as risk factors for stillbirth. In addition, there was an increased risk of stillbirth for babies delivered by CS rather than vaginal birth. This study also found a lack of adherence to guidelines regarding lack of monitoring FHR, the performance of CS on women presenting with no FHR at admission (25%) and lack of blood pressure measurement of the mother (64.5% of all).

### Stillbirth rate

The stillbirth rate of 22 stillbirths per 1000 total births found in this cohort did not fulfil ENAP’s goal of 12 stillbirths per 1000 total births in 2030 [23].

Maaløe et al. found a higher stillbirth rate of 59 per 1000 births in the only tertiary hospital in the Zanzibar Archipelago, Mnazi Mmoja Hospital [14]. Similar to our study, a large study from 2018 found a community-based stillbirth rate of 25.7 per 1000 total births on Pemba [6]. Additionally, the Ministry of Health of Zanzibar has, for the first time, reported a stillbirth rate for 2019 that corresponds to 27 stillbirths per 1000 total births in Pemba [12].

Other studies on stillbirth in Sub-Saharan Africa reported national stillbirth rates ranging from 20 stillbirths per 1000 total births in Uganda to 118 stillbirths per 1000 total births in Sierra Leone [7, 24, 25]. Most of these stillbirth rates were higher than what we identified in Pemba, which could be explained by differences in study sites, setting and the diseases dominating in different sites, such as malaria, HIV and syphilis, as well as the fact that not all women giving birth at the hospitals gave consent to participate in this study [2].

### Risk factors for stillbirth

Our study investigated pregnancy and obstetric risk factors of stillbirth and found that women with previous CSs were at increased risk of stillbirth. That was in line with a review from Sandall et al., where previous CSs were found to be associated with stillbirth [26]. However, one study from Tanzania did not associate previous CS with perinatal mortality [27]. The variance could be explained by the study by H. Litorp et al. being conducted at a university hospital, which could have a better quality of care and monitoring for vaginal birth after CS. Our study found that nearly all women attended at least one ANC visit, and half of the women attended the four visits as recommended.

In contrast to our findings, other studies and reviews showed that inadequate ANC coverage was associated with stillbirth [7, 28]. However, this could indicate that the quality of ANC in Pemba is not high enough to help prevent stillbirths. Gwako et al. found that low quality of ANC was associated with an increased risk of stillbirth [29]. Preeclampsia is a known risk factor for stillbirth, especially for antepartum stillbirth [2]. Similar to our study, studies from Zanzibar, a multi-country study from Sub-Saharan Africa, South Ethiopia, Tanzania, and Nigeria, found an increased risk of stillbirth associated with preeclampsia [16, 25]. Furthermore, hypertensive disorders in pregnancy are commonly described as a risk factor in several reviews [2, 28, 30, 31]. This indicates a need for further attention to women with preeclampsia and hypertensive disorders in pregnancy to prevent stillbirths. It should be noted, that obstetric history in this study was collected from the women, and therefore results on disorders like preeclampsia should be read with some caution.

Like studies from Ethiopia, Zanzibar, Tanzania and a multi-country study from Sub-Saharan Africa, we found an increased risk of stillbirth to be associated with meconium-stained amniotic fluid and slow FHR [7, 32, 33]. Meconium-stained amniotic fluid and slow FHR are known indicators of foetal distress leading to asphyxia and, ultimately death [34, 35]. A systematic review from 2018 by Reinebrant et al. likewise indicated that the most common causes of stillbirth in low-income countries were hypoxic peripartum causes, infection, antepartum haemorrhage and more than 50% were unexplained causes or other unspecified causes [36].

Breech and cephalic malpresentation were risk factors in two multi-country studies [7, 37], as well as in Nigeria and Northern Tanzania [16, 38]. Our study found that breech presentation or cephalic malpresentation was 17 times more likely in stillbirths than live births. These findings highlight that breech and cephalic malpresentation are essential to identify before birth to intervene timely and plan the birth.

Several other studies reported prolonged and obstructed labour and malpresentation as a risk factor for stillbirth [7, 32, 39, 40]. However, the definitions and factors associated with prolonged labour and obstructed labour varied across the studies and could not be directly compared with our findings. Our study found that rupture of membranes 18 h before birth was 2.5 more likely for women with a stillbirth. Prolonged labour increases the risk of foetal distress and, subsequently, foetal asphyxia along with an increased risk of intrapartum infection [41, 42].

CS was frequent for women giving birth to stillbirths in Ethiopia, Mozambique and East Africa, in line with our findings [15, 40, 43]. The higher rate of CSs in women with stillbirth could be explained by a response of healthcare workers on detection of foetal distress, thus leading to the decision of CS, but also with a possibility of delay in the performance of CS. However, other studies in Ethiopia and Tanzania found no association between CS and stillbirth [25, 32]. This could indicate that the decision on CS is not made timely or delays in time from the decision to the procedure. Nevertheless, unnecessary CS is a dangerous procedure in this low-resource setting with few doctors to conduct the procedure. CS, without indication, is placing the woman at unnecessary risk in both the present and future pregnancies. The absolute risk of maternal death increases, especially in low-income settings and in the following pregnancy, the risk of hysterectomy, abnormal placentation, uterine rupture, stillbirth and preterm birth increases significantly [26]. Risks are lower with elective CSs [26]. The overall CS rate was 8.1% on Pemba in 2019 [12]. According to a WHO statement, CS rates above 10–15% do not improve maternal or newborn health [44]. This study did not identify the reasons and decision-making surrounding CS, but this could be valuable information that could add further to the understanding of why the decision to conduct CS is made and what the consequences could be.

### Quality of care

Guidelines on pregnancy and childbirth from WHO and National guidelines provided from the Ministry of Health were available on each hospital. Adherence to clinical guidelines in our study was challenging, with FHR and blood pressures not being measured and CS performed in cases without detectable heart rate at admission. Recording blood pressure, foetal heart rate, and even more so, vaginal examination for filling in partographs takes time. The hospitals faced a shortage of healthcare workers, a key challenge to adequately respond to obstetric complications and provide high-quality care. Often a healthcare worker assisted multiple women in giving birth simultaneously, further challenging the quality of care and adherence to guidelines. A study from Zanzibar showed similar results with a lack of measurement of blood pressure, lack of FHR monitoring, CS on women with no FHR on admission and an uneven distribution of midwives per birth-giving woman [14]. A study from mainland Tanzania similarly found that FHR monitoring was not measured regularly [25]. Guidelines suggest that women with previous CSs should be monitored closely during labour and childbirth [45]. We found an increased risk of previous CS to be associated with stillbirth, supporting that women with previous CSs need close monitoring and close following of protocols [45, 46]. This further indicates that women with previous CSs are complicated to assist and monitor in this setting with limited human resources. Local adaptions of guidelines and proper use of them have shown significantly decreased stillbirth numbers in a study by Maaløe et al. [47]. The number of partographs included and collected in this study proved insufficient, and further analyses were discarded. Direct observations before the study also suggested that partographs were scarcely used, not filled out according to clinical guidelines, or filled out retrospectively and thus not used as a tool to support clinical decision-making. Maaløe et al. found, similar to this study that the clinical guidelines on the use of the partograph were not achievable due to workload [47]. If partographs were seen more as an obstacle for the healthcare workers than a tool for assessment and quality of care, this could explain our findings. It could be suggested that an intervention on partograph use could positively affect adherence to guidelines and stillbirth rates on Pemba.

### Strengths and limitations

To our knowledge, only very few studies on stillbirths have been conducted prospectively in district hospitals, even though more than half of the births in the Zanzibar Archipelago take place in district hospitals or lower-ranged facilities [12]. The strengths of this prospective study were its study design and setting, along with the extensive study population presented. Our stillbirth incidence was in line with the stillbirth rate reported by the Ministry of Health (MoH) Zanzibar for 2019 [12]. This supports the validity of our findings, although it should be noted that the official recording of stillbirths likely faces similar same reporting barriers as this study did. The higher stillbirth incidence among mothers that did not consent to the study (41/1000) suggests that both the stillbirth rate identified by the MoH and the rate found in our study might underestimate the true stillbirth burden.

Furthermore, sociodemographic characteristics of mothers were not significantly different between stillbirths and live births. Given the high homogeneity of the study population, this suggested that our data was valid. Finally, data quality was relatively high, with a limited amount of missing data.

However, the study has several limitations. Firstly, the period of data collection was only 11 weeks. A more extended study period could have included more stillbirths, thus making the statistics more robust. The ORs should be read with caution, given the wide confidence intervals. Since the study did not have enough power to generate more robust statistic results, the results should be seen as a suggestion in how risk factors could be associated with stillbirths. Secondly, there were difficulties separating the variables of preterm rupture of membranes and rupture of membranes 18 h before birth. Some questions in the questionnaire were not precisely worded for the healthcare workers. However, the two terms were separated when asking the mothers. All results on these questions were, however, significant, suggesting that both were valid risk factors. Thirdly, this study did not include all possible births on Pemba Island and in the hospitals. Even though the facility-based birthing rate has increased on the island, approximately 30% of women still give birth at smaller facilities or at home [12]. Considering the importance of skilled birth assistance to prevent morbidity and mortality in the perinatal timeframe, Pemba’s district hospitals are likely a safer place to give birth. Therefore, our stillbirth rate might underestimate the stillbirth burden and risk factors of pregnancy and childbirth outside the hospitals, but this was outside the study’s objective.

Furthermore, the stillbirth rate for women who did not consent to participate in the study was 41 per 1000 total births (29/671). Women with stillbirths were thus overrepresented among those who did not give consent or withdrew consent after childbirth. Finally, questions were answered by mothers and healthcare workers after the birth. This could induce a recall bias regarding the memory of the procedures and actions. Recall bias were especially a risk in collecting data from the mother postpartum regarding her pregnancy and labour. When data are collected retrospectively with a broader timeframe, the risk of recall bias increases. It was likely that some information from the mother was forgotten or misremembered. The questionnaire for the health worker was collected just after the birth, and the risk of recall bias was thus lower. In some of the questions asked, they could look in medical records to answer the questions regarding especially the prenatal period, thus lowering the recall bias.

### Perspectives on stillbirth prevention

Causes and risk factors associated with stillbirth are not routinely collected in many countries with high stillbirth rates, including Zanzibar [2]. Thus, an important opportunity to gain a deeper understanding of the magnitude of the stillbirth burden is missed. Furthermore, data on why stillbirths occur and how to prevent them is limited [12], making it hard to design appropriate interventions to prevent them from occurring. Studies have shown that local adaptation of clinical guidelines, involvement and training of healthcare professionals, and education of populations could positively affect stillbirth rates [11, 47]. A systematic review from 2011 further emphasised the importance of skilled birth attendance and emergency obstetric care [48]. This study showed that in a district hospital setting with skilled birth attendants and opportunity for emergency obstetric care, the stillbirth incidence was twice the stillbirth rate goal of 12 stillbirths per 1000 total births [23]. That indicates a need for further interventions on better quality of care before and during childbirth to reduce the stillbirth burden.

In this study, we identified risk factors to be associated with stillbirth. Data on risk factors and causes of stillbirths in low-and-middle-income countries are increasing, but interventions on how to decrease the number of stillbirths occurring are more limited. Thus, we find it important to discuss the possibilities of stillbirth prevention in relation to the risk factors identified in this study.

A review from E. Wastnedge et al. highlights the importance of condition recognition and diagnosis of high-risk pregnancies as a key approach to reducing stillbirths. This includes access and attendance to good quality ANC along with the establishment of community groups with home visits and peer counselling as key interventions [10]. They also reported the importance of health system strengthening and guideline implementation. Health system strengthening is often a complex challenge, particularly due to a lack of human resources and medical supplies. With proper involvement and investment of health facility workers along with local health ministries, an improvement is likely to be achievable. Proper guideline implementation is another crucial intervention that can reduce stillbirths. However, proper implementation of guidelines requires accessibility and ownership from the healthcare workers working with them to be fully achievable and implemented successfully. A Delphi statement by N. Housseine et al. also concluded that international guidelines were not in line with what was locally possible regarding the foetal heart rate monitoring [49]. Guidelines and protocols should be developed in close relationships with the health facility workers that use them, which ensures that the guidelines are more likely to be adhered to and are locally achievable [10, 50]. Education and training, in combination with implementing guidelines, show even better adherence. Training and education in guidelines, as well as monitoring and management of women and babies, are important tools to reduce not just stillbirths but maternal and antenatal morbidity and mortality as well. Training should be conducted frequently over time to fulfil the full potential and to ensure that even with a high turnover in staff, most staff have had the possibility for training [10].

Gondwe et al. found that audit of stillbirths could be an effective tool to increase the quality of care given, thus lowering stillbirths and neonatal deaths [51]. However, causes of death can be a challenging task in low-income countries with various classification systems and attribution of cause used [28]. Lawn et al. call for a simplified classification system that can be applied in low-income countries [2].

More acknowledgement of stillbirths as a critical indicator for the quality of care during childbirth, clinically feasible guidelines and training, strengthening of health systems and a feasible classification and audit system could be the first steps to move forward [3, 10, 49, 50].

## Conclusion

This study on Pemba Island, Tanzania, found a stillbirth rate in the cohort of 22 per 1000 total births at four district hospitals in a rural setting. A stillbirth rate of 27.5 stillbirths per 1000 total births was found during the study period. We found a significant correlation between stillbirths and previous caesarean section, preeclampsia, hospital location, low foetal heart rate, and decreased or no foetal movements. Breech presentation or cephalic malpresentation, prolonged labour, meconium-stained amniotic fluid and delivery by caesarean section were also associated with stillbirth. Furthermore, we found non-adherence to clinical guidelines regarding foetal heart rate measurements, the use of partographs, and unnecessary caesarean sections on women presenting with no foetal heart rate and blood pressure measurement on admission. Improved adherence to guidelines before and during childbirth, and hence higher quality of care, may be needed to reach the Every Newborn Action Plan’s goal of fewer than 12 stillbirths per 1000 total births in 2030.

## Electronic supplementary material

Below is the link to the electronic supplementary material.

**Additional file 1:** Appendix 1

## Data Availability

The data is available upon reasonable request from the corresponding author.

## List of abbreviations

OR: Odds ratio
CI: Confidence interval
CS: Caesarean section
FHR: Foetal heart rate
ANC: Antenatal care
GA: Gestation age
BW: Birth weight
