# Supplementary material for: Risk factors of stillbirths in four district hospitals on Pemba Island, Tanzania: a prospective cohort study
Source: BMC Pregnancy Childbirth. 2023 Apr 26;23:288. doi: 10.1186/s12884-023-05613-6 (PMC10131471; doi:10.1186/s12884-023-05613-6)
Supplement: Supplementary file 1 — Additional file 1: Appendix 1 [file 12884_2023_5613_MOESM1_ESM.docx]

**Appendix**

**Appendix 1** Pemba Island with an overview of hospitals, antenatal, delivery and postnatal bed capacity and human resources available at night. Annual delivery numbers are not shown to respect the local healthcare workers.
